# Single- and dual-band filtering-response power dividers embedded SIW filter with improved output isolation

**DOI:** 10.1038/s41598-017-03312-9

**Published:** 2017-06-13

**Authors:** Kaijun Song, Yu Zhu, Fan Zhang

**Affiliations:** EHF Key Laboratory of Science, School of Electronic Engineering, University of Electronic Science and Technology of China, Chengdu, Sichuan 611731 P. R. China

## Abstract

Two filtering-response power dividers embedded substrate integrated waveguide (SIW) filter are presented in this paper. By employing different SIW resonators, single- and dual-band filtering response are achieved, respectively. The even- and odd-mode method and the coupled resonator theory are applied to analyze the presented power dividers. In addition, a novel isolation technique using multiple isolation resistors is proposed to improve the output isolation performance of the filtering-response power divider with multiple resonators. To verify the proposed circuits, the single- and dual-band filtering-response power dividers are designed, fabricated and measured. Reasonable agreement between the simulated and measured results is achieved. Moreover, the measured output isolation of the fabricated dual-band filtering-response power divider with multiple resonators is about 20 dB, which demonstrates the theoretical predication.

## Introduction

With the rapid development of commercial and industrial electronic systems, various of passive or active radio frequency (RF) circuits have been presented^1–15^. Among them, the power dividers are widely used in circuits and subsystems due to the ability to divide/combine signals^5, 7–33^. Over the past decades, many attentions have been paid to various power dividers. Several waveguide-based power dividers/combiners, such as rectangular waveguide power dividers^7, 8^, coaxial waveguide power dividers^9–12^, ring-cavity power divider^13^, conical power divider^14^, and radial waveguide power dividers^15–17^, are widely investigated due to the high power capability and low insertion loss. Meanwhile, planar power dividers based on microstrip line^18–20^, slotline^21, 22^ and double-sided parallel-strip line (DSPSL)^23–25^ were also extensively studied due to their small size, low cost, and high integration capability.

Moreover, the conventional power dividers suffer from poor out-of-band rejection and frequency selectivity. To these problems, the power dividers with bandpass filtering response and harmonic suppression have been drawn more attentions in recent years^18, 22, 26–29^. The substrate integrated waveguide (SIW) is attractive for the design of microwave and millimeter-wave components^30–33^ due to its high Q factor, low cost and high integration capability. An eight-way radial SIW power divider is reported^31^. Compact size and low insertion loss were achieved at the same time. A wideband SIW power divider with high isolation is proposed^32^. The design method is derived from conventional waveguide magic T.

In this paper, two filtering-response power dividers embedded SIW filters are presented. By employing quarter-mode SIW (QMSIW) and half-mode SIW (HMSIW) resonators, single and dual-band response are achieved, respectively. At the same time, the even- and odd-mode analysis method and filter’s phase characteristic are introduced to provide the design procedure for the presented power dividers simultaneously. Reasonable agreements between simulated and measured results are achieved, which validates the validity of the design method.

## Methods

### Theoretical design

#### A. QMSIW resonator analysis

**Figure 1:**
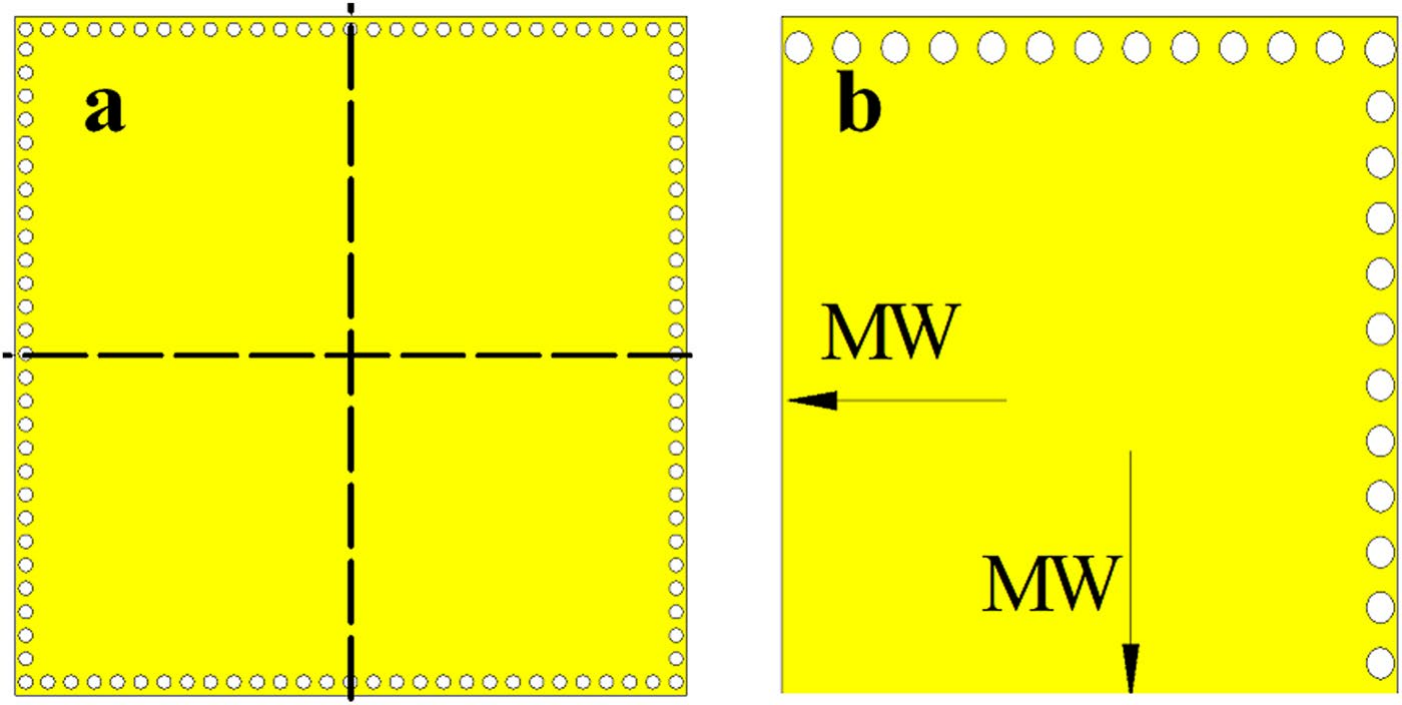
(**a**) SIW resonator (**b**) QMSIW resonator (MW: magnetic wall).

**Figure 2:**
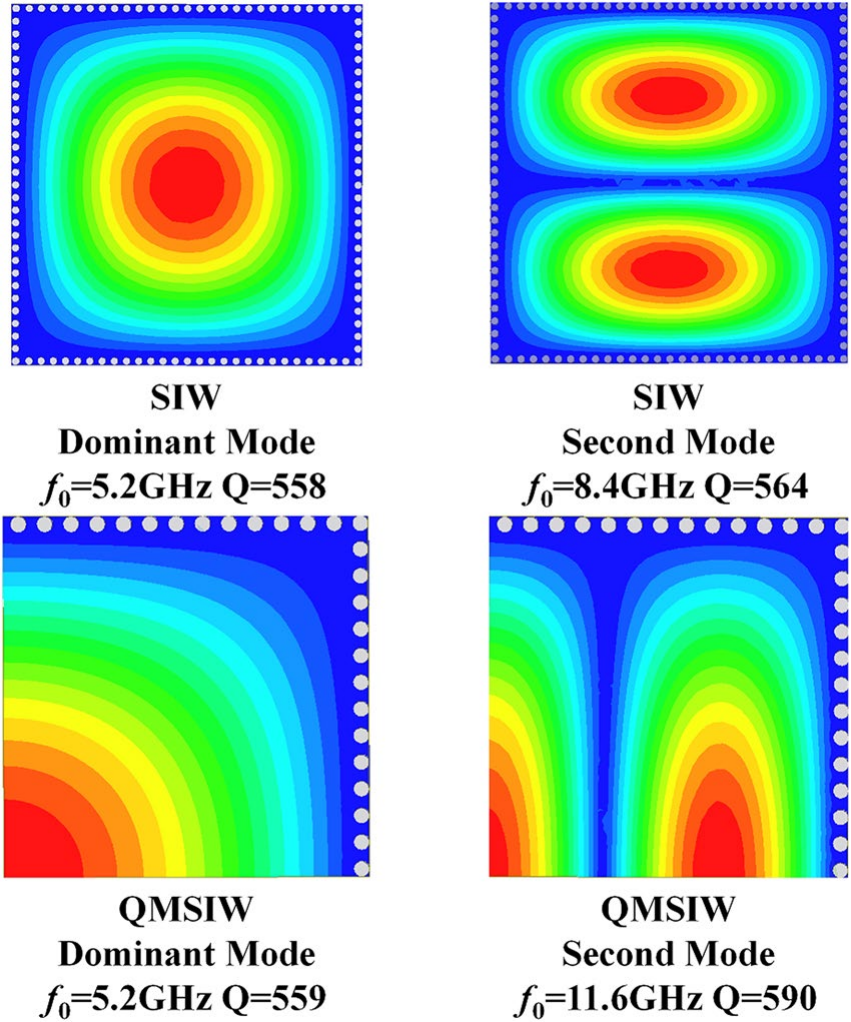
Comparison between SIW resonator and QMSIW resonator.

The conventional SIW resonator and QMSIW resonator are shown in Fig. 1. Compared with conventional SIW resonator (Fig. 1(a)), the size of the QMSIW resonator (Fig. 1(b)) is reduced by 75%. The resonant mode, resonant frequency, and unloaded Q factor of the two resonators are both shown in Fig. 2. It can be seen that the second mode (resonant frequency of 11.6 GHz) of the QMSIW resonator is TE103 mode, while that (resonant frequency of 8.4 GHz) of the SIW resonator is the TE102 mode. That is to say, the second-mode resonant frequency (11.6 GHz) of the QMSIW resonator is much farther away the dominant-mode resonant frequency than that (8.4 GHz) of the SIW resonator. So, the QMSIW resonator is more suitable for filters or power dividers to get wide stopband, high out-of-band attenuation, and compact size.

The resonant frequency of the dominant mode and second mode (TE103) can be obtained by

1 $${f}_{TE101}=\frac{c}{2\pi \sqrt{{\mu }_{r}{\varepsilon }_{r}}}\sqrt{{(\frac{\pi }{2{L}_{0}})}^{2}+{(\frac{\pi }{2{W}_{1}})}^{2}}$$

2 $${f}_{TE103}=\frac{c}{2\pi \sqrt{{\mu }_{r}{\varepsilon }_{r}}}\sqrt{{(\frac{\pi }{2{L}_{0}})}^{2}+{(\frac{3\pi }{2{W}_{1}})}^{2}}$$

Considering *L*_*0*_ = *W*_*1*_, when the resonant frequency of the dominant mode is chosen to 5.2 GHz, the dimensions of *L*_*0*_ = *W*_*1*_ = 10.9mm can be calculated from equation (1), which can be as the initial size of QMSIW resonator.

#### B. QMSIW filtering-response power divider

**Figure 3:**
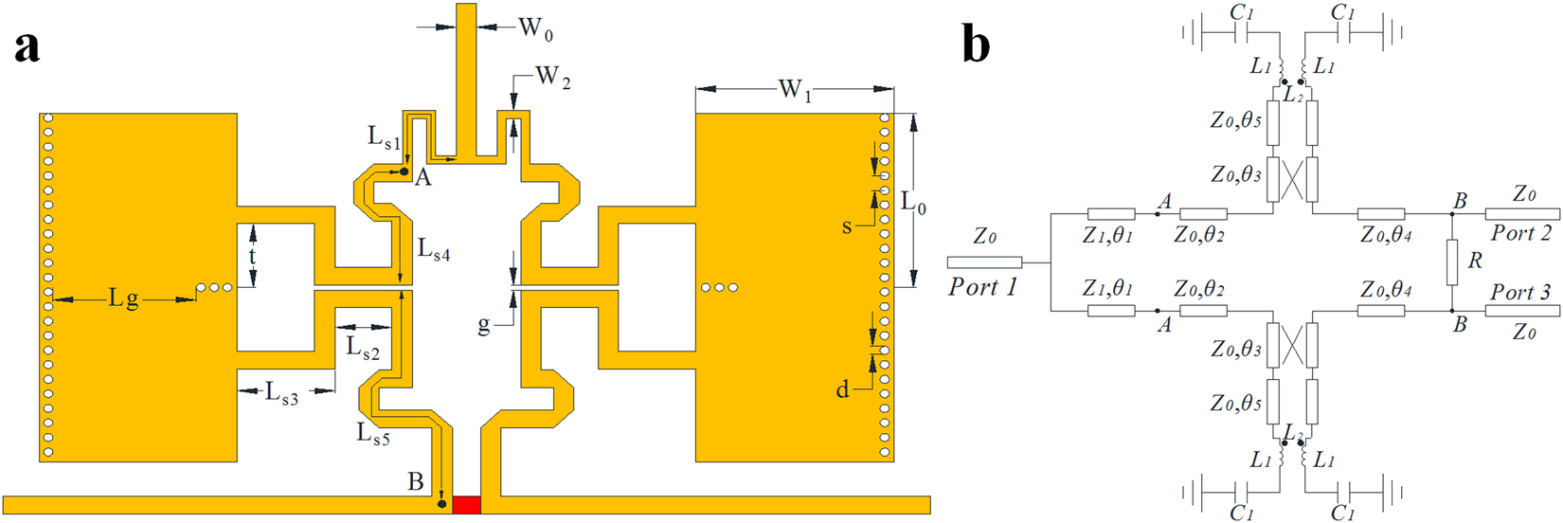
Presented single-band filtering QMSIW power divider (**a**) Configuration (**b**) Equivalent circuit.

Figure 3(a) shows the configuration of the presented single-band filtering-response power divider using QMSIW resonator, while Fig. 3(b) shows its equivalent circuit. The presented power divider is composed of four QMSIW resonators, λ/4 impedance transformers, isolation resistor, and input/output cross coupling structures. The input λ/4 impedance transformers can provide good input impedance matching, while the input/output cross coupling of QMSIW filter part can generate additional transmission zeros to improve the passband frequency selectivity and the out-of-band attenuation. Two QMSIW resonators for one way have been applied to achieve the desired bandpass filtering response.

**Figure 4:**
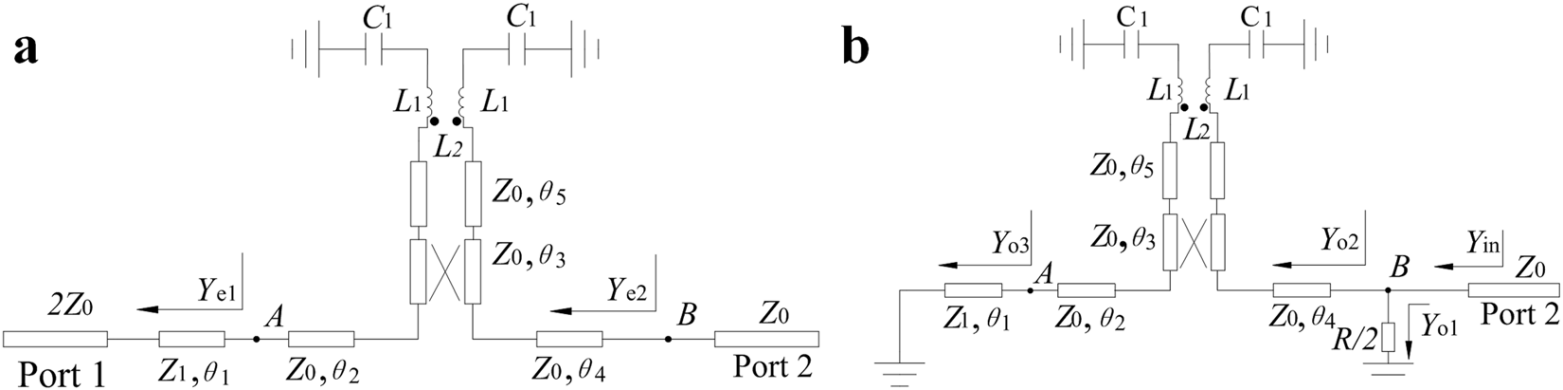
Equivalent circuit (**a**) even mode (**b**) odd mode.

Due to the symmetry of the circuit, the even- and odd-mode analysis technique can be applied to analyze the presented filtering power divider. The even- and odd-mode equivalent circuits are shown in Fig. 4(a) and (b).

The QMSIW resonator can be viewed as a LC series resonant circuit network^34^. In this case, the equivalent inductance and capacitance can be expressed as

3a $${L}_{{\rm{1}}}=\frac{{Z}_{q}\pi }{2{\omega }_{0}}$$

3b $${C}_{{\rm{1}}}=\frac{1}{{\omega }_{0}^{2}L}$$

where *Zq* is the characteristic impedance of the QMSIW, which is equal to the double of SIW ones, and can be calculated as follows

4 $${Z}_{q}=\frac{h}{{W}_{1}}\frac{{Z\text{'}}_{TEM}}{\sqrt{1-{(\frac{\lambda \text{'}}{4{W}_{1}})}^{2}}}$$

where *h* is the height of the substrate, *Z′*_*TEM*_ is the wave impedance and *λ′* is the operating wavelength in the dielectric, respectively.

**Figure 5:**
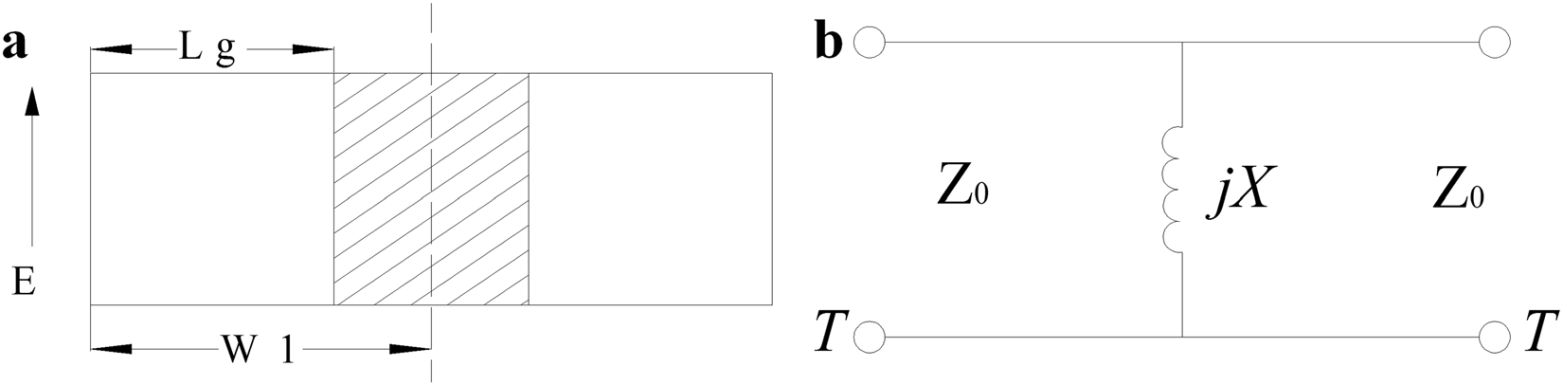
(**a**) Cross section view of SIW inductive window (**b**) Equivalent circuit.

The coupling window has been used between the two QMSIW resonators to get the suitable coupling coefficient, as shown in Fig. 3(a). Figure 5(a) and (b) show the simple circuit structure and the equivalent circuit of the SIW inductive window, respectively. Lg is the width of the inductive window. The equivalent inductance can be obtained^35^

5 $$\frac{X}{{Z}_{0}}=\frac{{W}_{1}}{2{\lambda }_{g}}[\mathrm{ln}(\frac{8k}{\pi {e}^{2}})+\frac{4}{27}{(\frac{2{W}_{1}}{\lambda })}^{2}],k <  < 1$$

6 $$k=\frac{{W}_{1}}{{W}_{1}-{L}_{g}}$$

**Figure 6:**
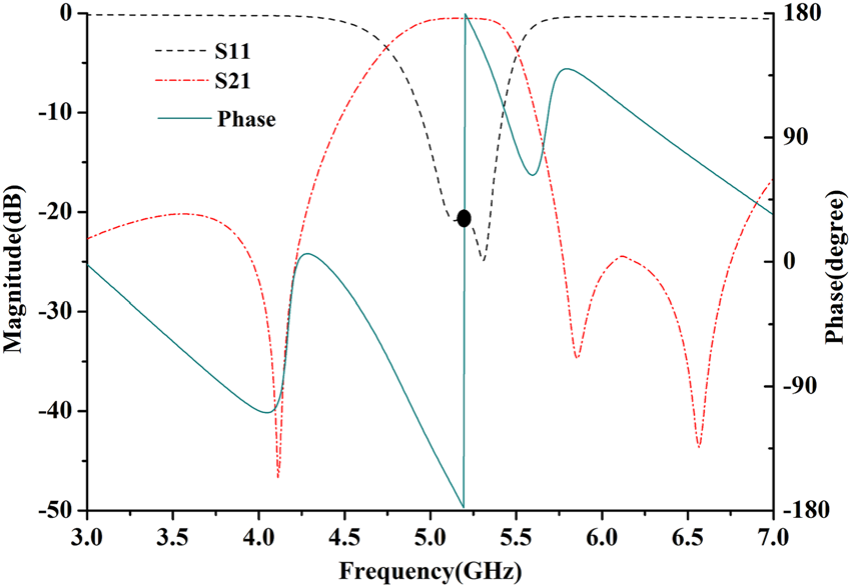
Simulated S-parameter magnitude and phase of the single band filter between.

For the even-mode circuit (Fig. 4(a)), the input admittance seen from the node A to port 1 is

7 $${Y}_{e1}={Y}_{1}\frac{{Y}_{0}/2+j{Y}_{1}\,\tan \,{\theta }_{1}}{{Y}_{1}+j{Y}_{0}\,\tan \,{\theta }_{1}/2}$$

where Y_0_ = 1/Z_0_ and Y_1_ = 1/Z_1_. If the phase shift of 180° between node A and node B is met (this condition can be met by adjusting the length of coupled-lines and the corresponding transmission lines (*θ*_*2*_ *, θ*_*3*_ *, θ*_*4*_), as shown in Fig. 6), then

8 $${Y}_{e1}={Y}_{e2}={Y}_{0}$$

When *θ*_1_ = λ/4, then

9 $${Y}_{1}={Y}_{0}/\sqrt{2}$$

In this case, the even-mode circuit can be viewed as a second-order bandpass filter with good input/output impedance matching. Then, the traditional coupled-resonator filter design method based on coupling coefficients and external quality factor can be employed directly to design the presented circuits.

For the odd-mode circuit (Fig. 4(b)), the input admittance at port 2 is

10 $${Y}_{in}={Y}_{o1}+{Y}_{o2}$$

where *Y*_o1_ = 2/*R* and *Y*_o2_ is the input admittance seen from the node B to the left filtering network (not including the shunt resistor R/2).

Similarly, when *θ*_1_ = λ/4, *Y*_o3_ = 0. As can be seen from Fig. 6, a phase shift of 180° (corresponding to a center frequency of 5.2 GHz) is introduced by the filter (between node A and node B). In this case, the filter (between node A and node B) can be then equivalent to a non-uniform transmission line with electronic length of λ/2. Then, the admittance *Y*_o2_ is

11 $${Y}_{o2}={Y}_{o3}=0$$

To get good input impedance matching, the following condition should be met

12 $${Y}_{in}={Y}_{0}$$

Then,

13 $$R=2{Z}_{0}$$

In this case, good impedance matching can be achieved at port 2 when *R* = 2*Z*_0_, which also implies that all of the power is delivered to the isolation resistor.

Then, the presented power divider can be simplified the design of a second-order bandpass filter between node A and node B. In this filter design, the Chebyshev response with 0.1-dB ripple is utilized and the desirable fractional bandwidth is 14.7%. g_0_ = 1, g_1_ = 1.1468, g_2_ = 1.3712. Moreover, the required external quality factor and coupling coefficient can be obtained^36^ as

14a $${Q}_{e}=\frac{{g}_{0}{g}_{1}}{FBW}$$

14b $$K=\frac{FBW}{\sqrt{{g}_{1}{g}_{2}}}$$

**Figure 7:**
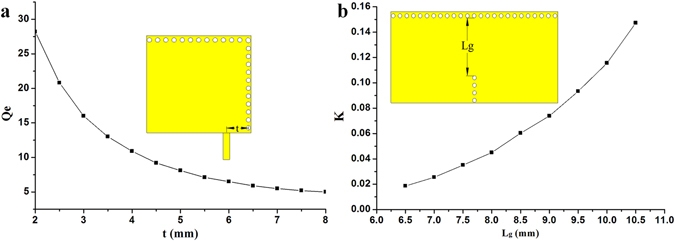
(**a**) Extracted external Q_e_ and (**b**) coupling coefficient *K*.

Figure 7 (a) and (b) show the external quality factor Q_e_ and coupling coefficient *K* with the varied circuit structural sizes, respectively. Then, the taper position t and the width L_g_ of the coupling inductive window can be determined according to the required external quality factor and coupling coefficient from Fig. 7.

In implementing the filtering power divider, the design procedures can be summarized as follows:

1. Determine the geometric parameters of QMSIW resonator to meet the given resonant frequency.
2. Find the coupling gaps between adjacent resonators to meet the given bandwidth and coupling coefficients.
3. Determine the taper position of the I/O resonator according to the given external quality factor.
4. Design the two-way power divider, and further complete the design of entire circuit.

#### C. Analysis of the dual-band filtering power divider using HMSIW with multiple isolation resistors

**Figure 8:**
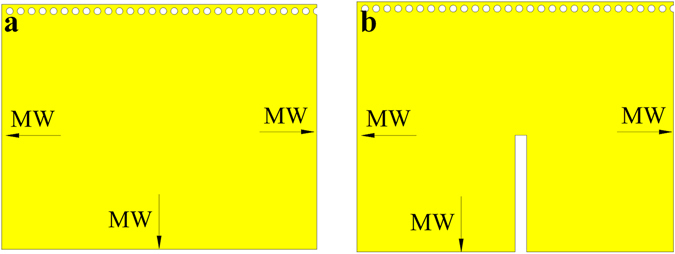
(**a**) Dual-mode HMSIW resonator without slot (**b**) Dual-mode HMSIW resonator with slot. MW—magnetic wall.

**Figure 9:**
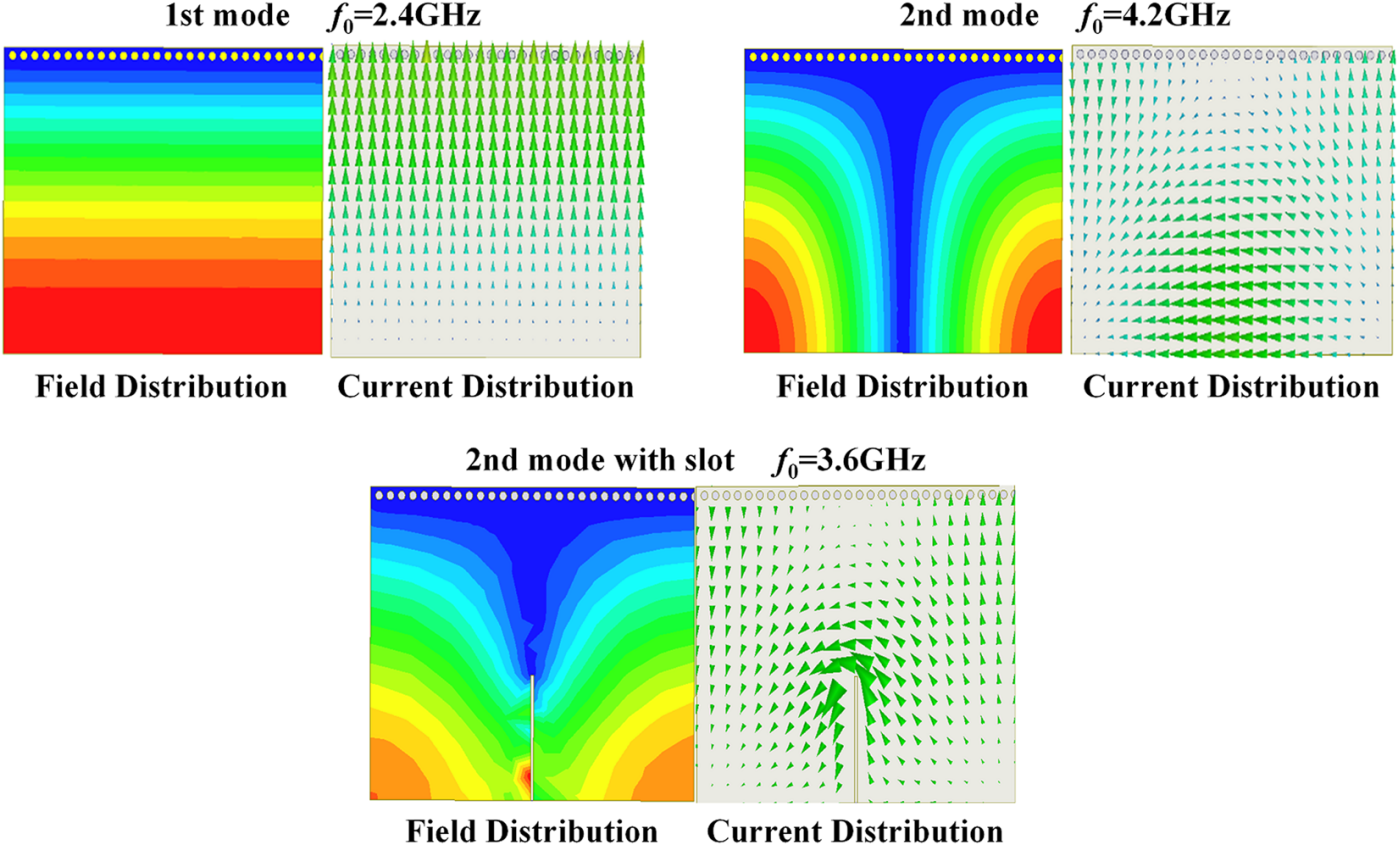
Field and current distribution of HMSIW resonator.

The proposed HMSIW resonator is shown in Fig. 8(a). Compared with conventional SIW resonator (Fig. 1(a)), the size of the HMSIW resonator (Fig. 8(a)) is reduced by 50%. The main advantage of the HMSIW resonator is the co-existence of the lower resonant mode—quasi-TEM mode^37^, which can be viewed as a short-circuit microstrip line resonator with electrical length of 90 degree, and the higher SIW cavity resonant mode—TE102 mode. Figure 8(b) shows the proposed HMSIW resonator with slot. The electric field and current distribution of the first and the second mode are shown in Fig. 9. It should be noted that the second resonant frequency can be tuned by changing the length of the slot embedded into the resonator^37^. Therefore, the dual-band power divider can be easily realized by replacing the QMSIW resonators in Fig. 3 with the dual-mode HMSIW resonators with slot.

The resonance frequency of quasi-TEM mode and TE102 mode can be calculated, respectively, as follows

15 $${f}_{TEM}=c/(4\ast {W}_{2}\sqrt{{\varepsilon }_{r}})$$

16 $${f}_{TE102}=\frac{c}{2\pi \sqrt{{\mu }_{r}{\varepsilon }_{r}}}\sqrt{{(\frac{\pi }{2{W}_{2}})}^{2}+{(\frac{\pi }{{L}_{1}})}^{2}}$$

**Figure 10:**
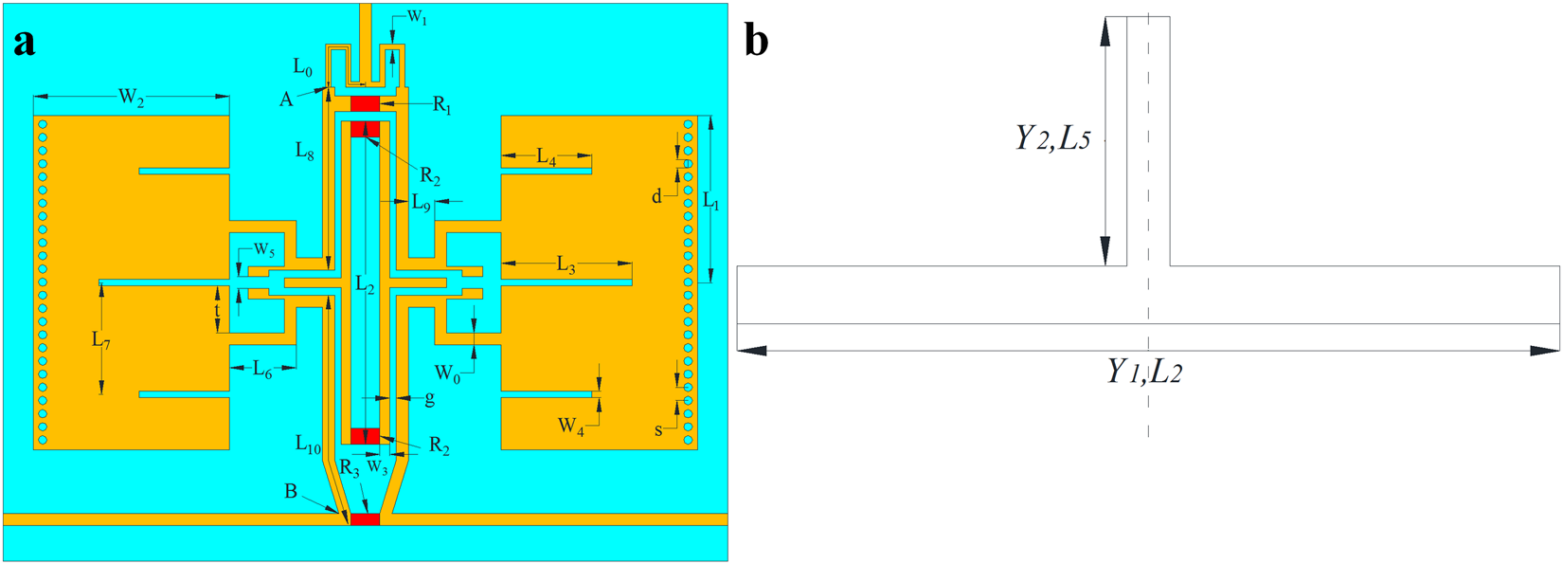
(**a**) Configuration of the dual-band HMSIW filtering power divider. (**b**) Dual-mode stub loaded resonator.

The configuration of the presented dual-band filtering power divider based on HMSIW resonators is shown in Fig. 10(a). When the center frequency of *f*_*TEM*_ = 2.3 GHz and *f*_*TE102*_ = 3.5 GHz of lower and upper passbands of dual-band HMSIW filter are chosen, the dimensions of *W*_*2*_ = 17.4mm and *L*_*1*_ = 22.9 mm (as shown in Fig. 10(a)) can be calculated from equation (15) and (16), which can be as the initial sizes for the design of HMSIW resonator.

To extend the operating bandwidth and improve the frequency selectivity, the dual-mode stub loaded resonator is embedded in the circuit (see Fig. 10(b)). The odd- and even-mode resonant frequencies *f*_*odd*_ and *f*_*even*_ can be expressed respectively as

17 $${f}_{{o}dd}=(2n-1)c/2{L}_{2}\sqrt{{\varepsilon }_{eff}}$$

18 $${f}_{even}=nc/({L}_{2}+2\ast {L}_{5})\sqrt{{\varepsilon }_{eff}}$$

where *f*_*odd*_ and *f*_*even*_ correspond to the center frequencies of the first and the second passband. Then, the lengths of *L*_*2*_ = 34.8mm and *L*_*5*_ = 5.4mm can be determined by the above equations (17) and (18). The dual-mode stub loaded resonator will introduce a transmission zero in the lower stopband, which can improve the frequency selectivity and out-of-band rejection. In addition, two small open stubs are used in the input and output ports of dual-band HMSIW filter parts to achieve input-output cross coupling.

To improve the isolation between the two output ports, four isolation resistors are employed in the presented dual-band power divider (see Fig. 10(a)). In the even-mode excitation, the symmetrical plane can be viewed as an electrical wall and the four isolation resistors have been open-circuited. In this case, the four isolation resistors can be omitted and the frequency response of the presented dual-band filtering power divider will not be changed by the four isolation resistors. However, in the odd-mode excitation, the symmetrical plane can be viewed as a magnetic wall and the current will flow through the four isolation resistors. In this case, the four isolation resistors can improve greatly the isolation between the two output ports.

**Figure 11:**
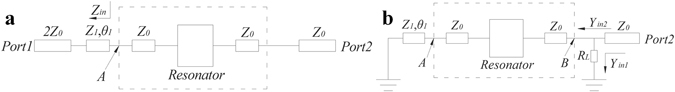
Equivalent circuit of dual-band filtering power divider before embed two dual-mode stub loaded resonator. (**a**) even mode. (**b**) odd mode.

**Figure 12:**
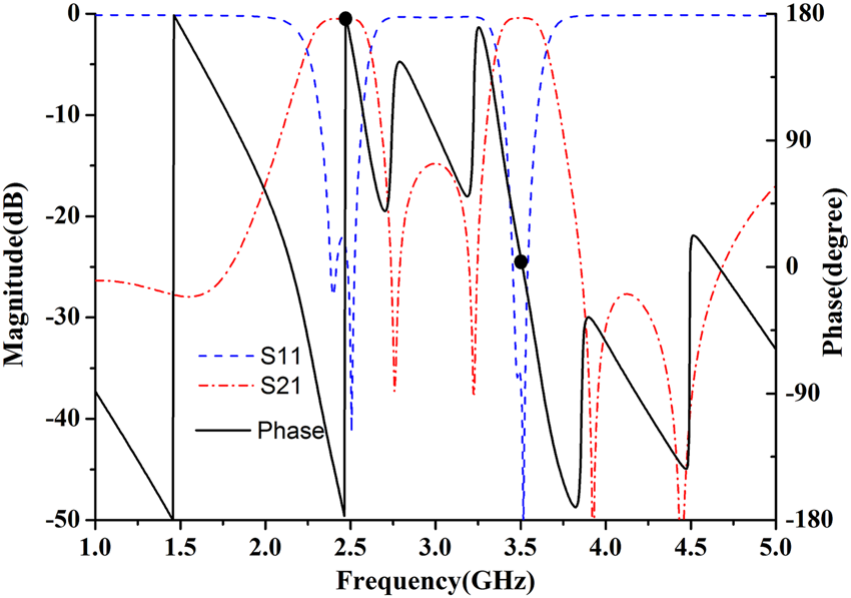
Simulated S-parameter of dual-band HMSIW filter before embed two dual-mode stub loaded resonator and phase between node A and node B.

Figure 11 shows the odd- and even-mode equivalent circuit before embed two dual-mode stub loaded resonator. Considering two passbands of the proposed circuit independent with each other, the circuit performance of each passband can be analyzed, separately. For two passbands, the even-mode equivalent circuit can be seen as a second-order filter of input and output matching. Under odd-mode exciting, for the first passband, the HMSIW resonator can be equivalent to a short-circuited microstrip-line resonator with quarter wavelength. When *θ*_1_ = λ/4, by adjusting the length of transmission lines (*L*_*6*_ *, L*_*9*_ *, L*_*10*_), the phase shift of 180° between node A and node B is met (as shown in Fig. 12), and the input admittance seen from the node B to input port is

19 $${Y}_{in2}=0$$

then

20 $$1/{{\rm{Z}}}_{0}={Y}_{in1}+{Y}_{in2}$$

where *Y*_in1_ = 2/*R*_*3*_, then

21 $${R}_{3}=2{Z}_{0}$$

For the second passband, HMSIW can be equivalent to a traditional waveguide resonator. Similarly, the input admittance seen from port 2 is

22 $$1/{{\rm{Z}}}_{0}={Y}_{in1}+{Y}_{in2}$$

By adjusting the length of transmission line (*L*_*8*_), the phase shift of 0° between node A and node B can be met (as shown in Fig. 12). Then, the entire filter network can be seen as a non-uniform transmission lines with half-wavelength. When *θ*_1_ = λ/4, then, *Y*_*in2*_ = 0, the isolation resistor *R*_*3*_ also meet

23 $${R}_{3}=2{Z}_{0}$$

(21) and (23) provides a simple guideline in the selection of *R*_*3*_.

Moreover, it can be seen the input circuit including isolation resistors R_1_ is similar with the Wilkinson power divider, then

24 $${R}_{1}=2{Z}_{0}$$

**Figure 13:**
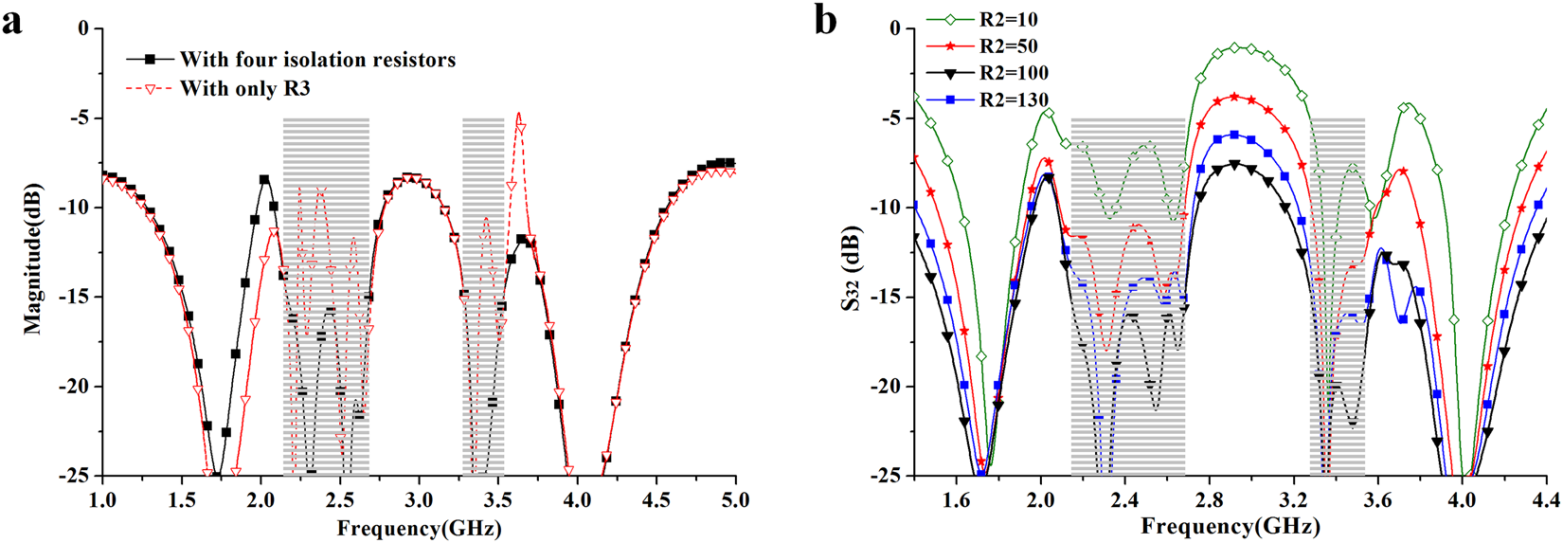
(**a**) Comparison of isolation performance with only R_3_ and four isolation resistors. (**b**) Isolation performance with different R_2_.

The comparison of isolation performance with only R_3_ and four isolation resistors are illustrated in Fig. 13(a). It can be seen that the dual-band isolation performance are improved greatly when the four isolation resistors are all applied to the presented power divider. Besides, Fig. 13(b) shows the dual-band isolation performance with different value of the isolation resistor R_2_. It can be seen that the isolation within the two passbands can be greatly improved with the increasing of R_2_. The good isolation within them can be achieved when R_2_ is equal to 100 ohm. Herein, R_1_ = R_2_ = R_3_ = 100 ohm.

Similarly, the design procedures of the dual-band power divider based on HMSIW resonators can be summarized as follows:

1. Determine the geometric parameters of HMSIW resonator to meet the given resonant frequency.
2. Find the coupling gaps between adjacent resonators to meet the given bandwidth and coupling coefficients.
3. Determine the taper position of the I/O resonator according to the given external quality factor.
4. Determine the geometric sizes of the stub loaded resonator to meet the desired resonant frequency.
5. Design the two-way power divider, and further complete the design of entire circuit.

## Results

### Results of the single-band QMSIW power divider

**Figure 14:**
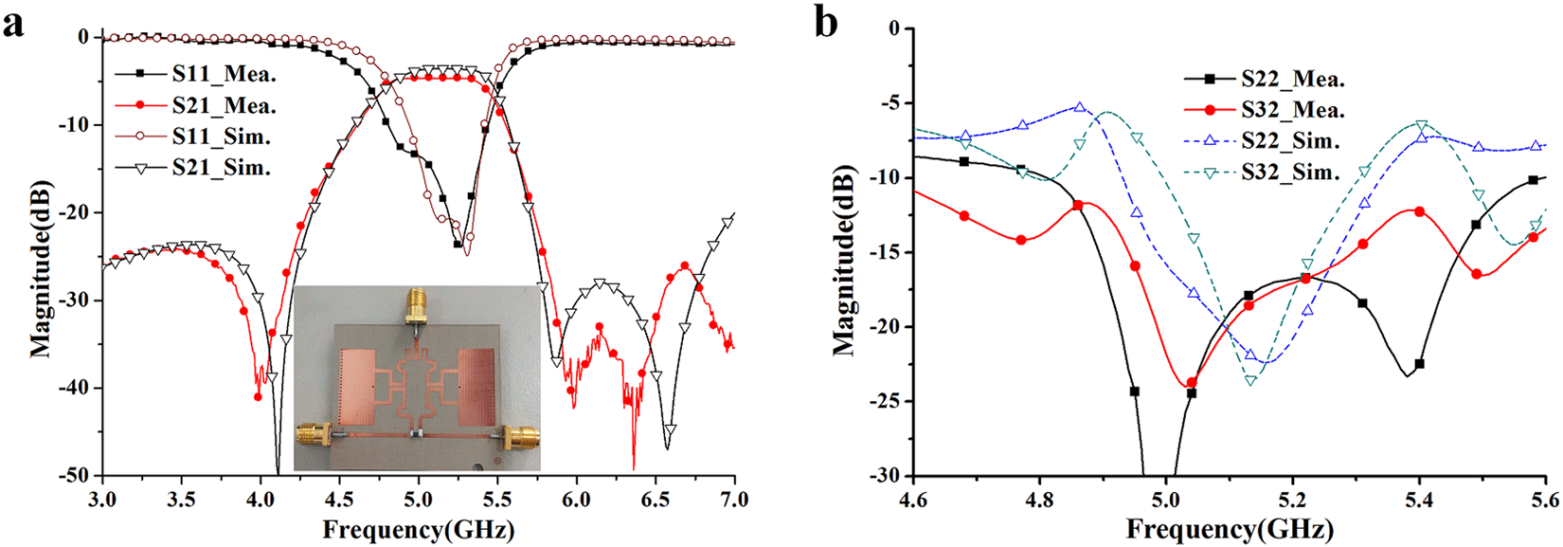
Photograph and results of the fabricated power divider. (**a**) Simulated and measured S_11_ and S_21_ (**b**) Output return loss S_22_ and isolation S_32_.

Based on the analysis above, the single-band QMSIW power divider is designed, simulated and optimized by HFSS 15, and fabricated on a substrate with relative dielectric constant of 3.5, thickness of 0.508mm, and loss tangent of 0.0018. The fabricated filtering-response power divider is shown in Fig. 14(a). Final optimized dimensions are listed in Table 1. The total size is about 1.5λ_g_ × 1.6λ_g_.

**Table 1:** Dimensions of the single-band power divider (unit: mm).

W_0_	W_1_	W_2_	L_0_	Ls_1_	Ls_2_	Ls_3_
1.11	11	0.5	11	9.1	3.14	5.36
Ls_4_	Ls_5_	L_g_	d	s	t	g
14.5	16.5	8.9	0.5	0.8	4	0.3

The simulated and measured results of the fabricated QMSIW power divider are shown in Fig. 14. The measured input return loss is greater than 13 dB from 4.9 to 5.4 GHz, while the simulated one is greater than 15 dB from 5 to 5.4 GHz, as shown in Fig. 14(a). The measured insertion loss is around 1.6 dB (the 3 dB power division loss is not included) from 4.9 to 5.4 GHz, while the simulated one is less than 1 dB from 4.95 GHz to 5.4 GHz. In addition, the out-of-band rejection level is more than 24 dB at the lower stopband (from 0 to 2.3 GHz) and 26 dB at the upper stopbands (from 5.8 to more than 7 GHz). The output return loss and isolation between the two output ports are shown in Fig. 14(b). The measured output return loss |S_22_| is around 17 dB over the operating frequency band, while the measured isolation |S_32_| between the port 2 and port 3 is about 15 dB. The measured results show reasonable agreement with the simulated ones. The increased insertion loss is due to the fabrication and assembly error, the loss of the additional microstrip line to coaxial type-SMA connector transition, dielectric constant error, loss tangent error, and measurement error. These were not included in the simulations. The difference between measured and simulated output return loss and isolation (as shown in Fig. 14(b)) can be attributed to the inaccuracies of relative position in assembling isolation resistor, the loss of the additional microstrip line to coaxial type-SMA connector transition, dielectric constant error, and measurement error. These were also not included in the simulations.

### Results of the fabricated dual-band HMSIW power divider with multiple isolation resistors

**Figure 15:**
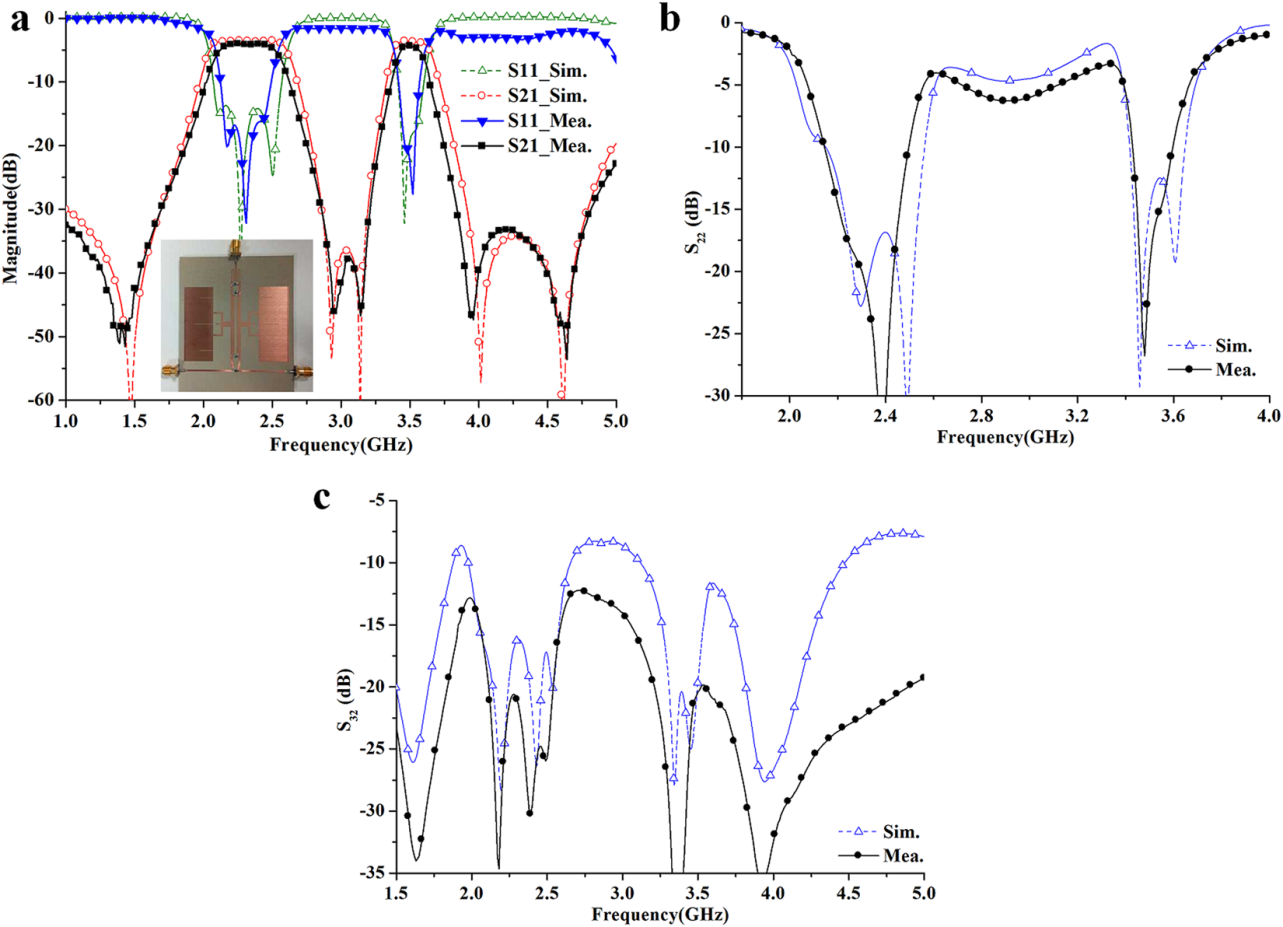
Simulated and measured results of the fabricated power divider (**a**) S_11_, S_21_, and photograph of the fabricated power divider (**b**) S_22_ (c) S_32_.

According to the above analysis and design method, the presented HMSIW dual-band filtering power divider with multiple isolation resistors is designed, simulated and fabricated. Simulations and optimizations are also accomplished by HFSS. The substrate of the dual-band filtering power divider is the same as that of the above single-band filtering power divider. Photograph of fabricated dual-band filtering power divider is shown in Fig. 15(a). The final dimensions are listed in Table 2.

**Table 2:** Dimensions of dual-band power divider (unit: mm).

W_0_	W_1_	W_2_	W_3_	W_4_	W_5_	L_0_
1.11	0.5	18	0.9	0.2	0.5	5.36
L_1_	L_2_	L_3_	L_4_	L_5_	L_6_	L_7_
23	40.8	12	8.3	4.8	6.11	3.3
L_8_	L_9_	L_10_	g	t	d	s
24.9	4.8	28.9	0.24	6.35	0.5	0.8

The simulated and measured results of the fabricated dual-band HMSIW power divider with multiple isolation resistors are shown in Fig. 15. The measured lower and upper passbands of the fabricated dual-band HMSIW power divider are centered at 2.3 GHz and 3.5 GHz, with fractional 1-dB bandwidths of 17.4% and 5%, respectively, as shown in Fig. 15(a). The measured lower- and upper-passband input return losses are greater than 16 dB from 2.15 to 2.45 GHz and 18 dB from 3.45 to 3.55 GHz, respectively. Moreover, the measured minimum insertion losses of the lower and upper passbands are 1.2 dB (the 3 dB power division loss is not included) and 1.6 dB, respectively, while the simulated ones are all greater than 0.55 dB within the two passbands. In particular, five transmission zeroes can be observed on both sides of the two passbands. The transmission zero located in the lower stopband is introduced by the dual-mode stub loaded resonator, while the others are generated by the cross coupling (the open coupling stub with gap W5 in Fig. 10(a)) between the input and output ports of the dual-band HMSIW filter parts. It can be seen that the frequency selectivity and out-of-band rejection are improved greatly by the five transmission zeros. The out-of-band rejection level is all more than 30 dB at the lower, middle, and upper stopbands.

The output return loss S_22_ and isolation S_32_ between the two output ports are shown in Fig. 15 (b) and (c). The measured |S_22_| is greater than 15 dB over the two passbands, while the measured isolation |S_32_| is also greater than 20 dB over the two passbands. Similarly, the increased insertion loss is also due to the fabrication and assembly error (including the inaccuracies of relative position in assembling multiple isolation resistors), the loss of the additional microstrip line to coaxial type-SMA connector transition, dielectric constant error, loss tangent error, and measurement error. These were not included in the simulations. Moreover, the comparison of the presented dual-band filtering power divider with some prior designs is summarized in Table 3.

**Table 3:** Comparison with some prior filtering power dividers.

References^18, 23, 25, 26, 29, 38, 39^	Band	15dB(S_11_) FBW	IL (dB)	Isolation (dB)	ORL (dB)	TZ	FS	BFR
[18]	2	8%/7.4% at 10dB((S_11_)	0.8/0.9	>12	10	3	moderate	yes
[23]	2	10%/4%	1/1.3	15	12	3	bad	no
[25]	1	100%	1.2	15	15	0	bad	no
[26]	1	3.5%	1.4	>30	15	0	bad	yes
[29]	1	4%	3	20	10	0	bad	yes
[38]	1	1%	2	>25	>20	0	bad	yes
[39]	1	1.8%	0.16	>26	>18	0	bad	yes
This work	2	17.4%/5%	1.2/1.6	20	15, 15	5	good	good

FBW: fractional bandwidth; IL: Insertion Loss; ORL: output return loss; TZ: transmission zero; FS: Freq. selectivity; BFR: bandpass filtering response.

## Discussion

Two types of filtering-response power dividers have been presented in this paper. The characteristics of the QMSIW resonator and HMSIW resonator are analyzed. The presented two power dividers are fabricated to verify the proposed analysis and design method. The simulated and measured results show reasonable agreement with each other. It can be seen that the presented power dividers have the advantages of good frequency selectivity, good input and output impedance matching, acceptable isolation performance, and low cost, which make it very competitive in the practical system applications.
